# Diagnosing and managing work‐related mental health conditions in general practice: new Australian clinical practice guidelines

**DOI:** 10.5694/mja2.50240

**Published:** 2019-06-24

**Authors:** Danielle Mazza, Samantha P Chakraborty, Bianca Brijnath, Heather Nowak, Cate Howell, Trevor Brott, Michelle Atchison, David Gras, Justin Kenardy, Richard Buchanan, Seyram Tawia

**Affiliations:** ^1^ Monash University Melbourne VIC; ^2^ National Ageing Research Institute Melbourne VIC; ^3^ Mental Health Australia Canberra ACT; ^4^ Royal Australian College of General Practitioners Melbourne VIC; ^5^ Western Industrial Screening and Accident Clinic Melbourne VIC; ^6^ Royal Australian and New Zealand College of Psychiatrists Melbourne VIC; ^7^ Royal Australasian College of Physicians Melbourne VIC; ^8^ University of Queensland Brisbane QLD; ^9^ Australian Psychological Society Melbourne VIC; ^10^ Office of Industrial Relations Queensland Government Brisbane QLD; ^11^ Comcare Melbourne VIC

**Keywords:** Guidelines as topic, General practice, Anxiety disorders, Depressive disorders, Post‐traumatic stress disorder, Workers’ compensation

## Abstract

**Introduction:**

In Australia, mental health conditions (MHCs) arising from workplace factors are a leading cause of long term work incapacity and absenteeism. While most patients are treated in general practice, general practitioners report several challenges associated with diagnosing and managing workplace MHCs.

This guideline, approved by the National Health and Medical Research Council and endorsed by the Royal Australian College of General Practitioners and the Australian College of Rural and Remote Medicine, is the first internationally to address the clinical complexities associated with diagnosing and managing work‐related MHCs in general practice.

**Main recommendations:**

Our 11 evidence‐based recommendations and 19 consensus‐based statements aim to assist GPs with:

- the assessment of symptoms and diagnosis of a work‐related MHC;
- the early identification of an MHC that develops as a comorbid or secondary condition after an initial workplace injury;
- determining if an MHC has arisen as a result of work factors;
- managing a work‐related MHC to improve personal recovery or return to work;
- determining if a patient can work in some capacity;
- communicating with the patient's workplace; and
- managing a work‐related MHC that is not improving as anticipated.

**Changes in management as result of the guideline:**

This guideline will enhance care and improve health outcomes by encouraging:

- the use of appropriate tools to assist the diagnosis and determine the severity of MHCs;
- consideration of factors that can lead to the development of an MHC after a workplace injury;
- more comprehensive clinical assessments;
- the use of existing high quality guidelines to inform the clinical management of MHCs;
- consideration of a patient's capacity to work;
- appropriate communication with the workplace; and
- collaboration with other health professionals.

In Australia, mental health conditions (MHCs) arising as a result of work factors are a leading cause of long term work incapacity and absenteeism.^1^ According to workers’ compensation claims data, people with an accepted claim for a work‐related mental injury take three times longer to return to work compared with the median time away from work for all claims.

General practitioners play a crucial role in assessing and diagnosing patients with MHCs arising from the workplace and in assisting these patients to manage their condition and meet personal recovery goals.^2^

Until now, there have been no clinical practice guidelines that address the clinical complexities associated with diagnosing and managing work‐related MHCs in general practice. To the best of our knowledge, this guideline is the first internationally to describe the best available evidence on diagnostic and management options for patients with work‐related MHCs.^3^

The guideline focuses on MHCs that may have arisen as a result of work, such as depression, anxiety, post‐traumatic stress disorder (PTSD), acute stress disorder, adjustment disorder and substance use disorder,^3^ and builds upon key principles articulated in the Health Benefits of Good Work consensus statement,^4^ and the Fifth National Mental Health and Suicide Prevention Plan, which emphasises that “consumers and carers have vital contributions to make and should be partners in planning and decision‐making”.^5^ Underlying the clinical recommendations are also two key principles: that GPs provide care within their expertise, knowledge and capabilities, and that GPs ensure that culturally and linguistically diverse patients and young people receive appropriate care throughout their recovery, with GPs working with these patients and other relevant practitioners to determine the best care options for patients.

## Methods

The guideline^3^ was developed according to the National Health and Medical Research Council (NHMRC) standards^6^ to ensure that appropriate governance structures, management of conflicts of interest, and methodological rigour were in place. An independent guideline development group oversaw the guideline development process. Members included three content experts, a consumer with a lived experience of a work‐related MHC, a GP from the Royal Australian College of General Practitioners (RACGP), an occupational physician from the Royal Australasian College of Physicians, a psychiatrist from the Royal Australian and New Zealand College of Psychiatrists, a state‐based policy maker, and a compensation scheme representative. A steering group, comprising representatives from the project sponsors, was established to ensure completion of the guideline according to milestones and to guide dissemination. The key clinical questions that are addressed in this guideline were based on clinical dilemmas identified by practising GPs and were developed using a qualitative research approach to incorporate views from guideline end users (ie, GPs, psychiatrists and compensation scheme workers).

A systematic review of the literature was carried out for each clinical question to build the evidence base for the development of the guideline recommendations. This body of evidence was given a strength rating according to the Grading of Recommendations Assessment, Development and Evaluation (GRADE) approach.^7^ A draft of the guideline was available for public consultation between 15 January and 15 March 2018 to elicit feedback, and revisions were made after public consultation. Two independent methodologists reviewed the guideline to consider its alignment with the Appraisal of Guidelines for Research and Evaluation (AGREE II) checklist, and content experts assessed it before the NHMRC approved the recommendations and the RACGP and the Australian College of Rural and Remote Medicine endorsed the guideline.

## Recommendations

The full guideline is accessible at http://www.monash.edu.au/work-related-mental-health-guideline. Below is an overview of the recommendations, consensus statements and practice points most likely to influence practice change.

### What tools can assist a general practitioner in diagnosing and assessing the severity of a mental health condition?

An accurate diagnosis is an essential step towards recovery for patients with an MHC. A diagnosis can be used to alleviate patient concerns about their signs and symptoms and provide patients with a rationale for how these symptoms emerged, and to consider and select optimal management strategies for the patient.^8^ For those patients who decide to submit a claim for compensation through a workers’ compensation scheme, a clearly stated diagnosis on the certificate of capacity — that is, the form that is submitted by an injured worker who is seeking compensation through a workers’ compensation scheme — can assist with an efficient assessment of the claim.^9^ For a comprehensive clinical assessment of a patient with a possible work‐related MHC, tools can assist GPs to make an accurate diagnosis and assess the severity of the condition (Box 1).

Box 1 **Recommendations in response to the question “What tools can assist a general practitioner in diagnosing and assessing the severity of a mental health condition?”**

**Table:** 

	Evidence level
For workers with symptoms of mental health conditions, a GP should use: ▶ the Patient Health Questionnaire‐9 (PHQ‐9)^10^ to assist in making an accurate diagnosis of depression and assess its severity; ▶ either the Generalized Anxiety Disorder 7‐item (GAD‐7) scale^11^ or the Depression Anxiety Stress Scales (DASS)^12^ to assist in making an accurate diagnosis of an anxiety disorder, and the Post‐traumatic Stress Disorder (PTSD) Checklist — Civilian Version (PCL‐C)^13^ to assist in making an accurate diagnosis of PTSD and assessing its severity; ▶ the Alcohol Use Disorders Identification Test (AUDIT),^14^ the Severity of Alcohol Dependence Questionnaire (SADQ),^15^ or the Leeds Dependence Questionnaire (LDQ),^16^ to assist in making an accurate diagnosis of an alcohol use disorder and assess its severity; and ▶ the LDQ^16^ to assist in making a diagnosis of substance use disorders and assess their severity	GRADE: Strong; Evidence: High
Adjustment disorder implies a level of distress greater than would otherwise be expected after a certain event. It is sometimes diagnosed when other psychiatric illnesses such as major depression and anxiety have been excluded, and is time‐limited. There are no recommended tools for diagnosing adjustment disorder or assessing its severity in general practice. A GP may consider use of the DASS^11^ to assess levels of patient distress and the World Health Organization Disability Assessment Schedule 2.0 (WHODAS 2.0)^17^ to assess levels of functional impairment	Consensus‐based recommendation
Tools should be used alongside a comprehensive clinical assessment, which includes consideration of cultural issues	Practice point
The advice of a specialist mental health clinician (eg, psychiatrist or clinical psychologist) should be sought by a GP if they are experiencing difficulties in diagnosis	Practice point

GRADE = Grading of Recommendations Assessment, Development and Evaluation.

The tools described in Box 1 should be used to support a comprehensive clinical assessment, which should be guided by the *Diagnostic and statistical manual of mental disorders* (DSM) 5 criteria for each condition.^18^ Australian GPs commonly use the Kessler Psychological Distress Scale (K10)^19^ or the Depression Anxiety Stress Scales (DASS) 21,^20^ to assist in making a diagnosis of depression. However, we did not identify any studies that described their validity or reliability in assessing depression by GPs in a work context. The Patient Health Questionnaire‐9 (PHQ‐9)^10^ and the DASS^12^ had high validity, with the PHQ‐9 also demonstrating high reliability for identifying depression. We therefore recommend using the PHQ‐9 for the assessment of depression and its severity for patients with symptoms indicative of depression that may have arisen from work.

While the systematic literature review provided strong support for use of the PTSD Checklist — Civilian Version (PCL‐C)^13^ to assist in the diagnosis of PTSD and assessment of its severity, a newer version of the PCL‐C, known as the PTSD Checklist for DSM‐5 (PCL‐5)^21^ is now available. The PCL‐5 is a 20‐item tool that assesses the 20 symptoms of PTSD described in the DSM‐5 — compared with the PCL‐C, which was validated against the symptoms of PTSD described in the DSM‐IV. The PCL‐5 has been validated in the military veteran population but requires further validation in the general patient population. Despite its limited validation, the PCL‐5 may be used to assist in making the clinical diagnosis of PTSD based on the DSM‐5.

### What would suggest that the patient is developing a comorbid or secondary mental health condition?

Patients with a substantial or chronic physical condition are twice to thrice more likely to develop depression compared with people who have no comorbidities,^22^ and the incidence of comorbid psychological conditions is well established.^23^ Not surprisingly, a significant number of patients who sustain a physical or psychological injury develop a comorbid or secondary MHC.^24^ Box 2 provides advice about patient and work factors that the GP may consider to assist in the detection of a developing MHC in patients with a physical or psychological workplace injury.

Box 2 **Recommendations in response to the question “What would suggest that the patient is developing a comorbid or secondary mental health condition?”**

**Table:** 

	Evidence level
For patients with a primary physical or psychological work‐related injury, a GP may consider the following factors to assist in the early detection of a comorbid or secondary mental health condition:	
Patient‐related factors: ▶ greater pain intensity, where physical injury was the precursor to the mental health condition; ▶ insomnia, low mood, anhedonia and suicidal thoughts; ▶ any existing substance misuse; ▶ a chronic physical health problem; ▶ lower self‐efficacy (ie, the capacity for one to cope with difficult demands through one's own effort); ▶ lack of social support and personal relationship status (ie, relationship problems); ▶ past experience of and response to treatments; ▶ past history of depression; ▶ perception of injustice of the compensation claim process;	GRADE: Weak; Evidence: Low
▶ pre‐existing depressive disorder or other anxiety disorder; and	Consensus‐based recommendation
▶ any other existing medical condition	Consensus‐based recommendation
Work‐related factors: ▶ job strain; ▶ failure to return to work after an injury	GRADE: Weak; Evidence: Low

GRADE = Grading of Recommendations Assessment, Development and Evaluation.

GPs may also work with other providers involved in facilitating the patient's recovery who have the expertise and judgement to assist in the assessment of a secondary MHC following an initial work‐related injury. For instance, all patients with an accepted claim have access to a workplace rehabilitation provider.^25^ Where the workplace rehabilitation provider has expertise in MHCs (eg, as a registered psychologist), they can collaborate with the GP to identify work‐related factors that can contribute or are contributing to the development of a secondary MHC.

For patients in rural and remote Australia, some of the factors described in Box 2 may be exacerbated. This may be due to the limited availability of mental health professionals, particularly those with expertise in work‐related injury. It is therefore important for GPs practising in rural and remote regions to be vigilant about the development of comorbid or secondary MHCs.

It is also important to note that the recommendations listed in Box 2 should not be used to indicate compensable status.

### Has the mental health condition arisen as a result of work?

The GP's opinion about whether an MHC has arisen out of work has significant implications on a patient's recovery and claim for compensation, but making such a determination is challenging for Australian GPs.^1^ Difficulties may arise because the factors that can cause or aggravate an MHC can be complex to investigate and authenticate. Furthermore, GPs have reported challenges in distinguishing between MHCs that developed as a result of work‐related stress and those that relate to a pre‐existing mental illness. Box 3 provides recommendations to assist GPs in making a determination about whether work factors are likely to have contributed to the presenting MHC. GPs may also work with other health care professionals with expertise in MHCs in making this determination.

Box 3 **Recommendation in response to the question “Has the mental health condition arisen as a result of work?”**

**Table:** 

	Evidence level
The assessment of whether a diagnosed mental health condition has arisen as a result of work should be made on the basis of: a comprehensive clinical assessment; consideration of factors such as pressures, events and/or changes in the workplace and the temporal relationship between these factors and symptom onset; and consideration of whether the mental health condition is consistent with the description of how the condition arose	Consensus‐based recommendation

In the absence of a validated tool to assist GPs in making this assessment, a clinical judgement about the work‐relatedness of an MHC can be made by undertaking a thorough history of the condition, and undertaking detailed consideration of the person's circumstances and current and past medical history. Key aspects of the GP's clinical judgement will involve the GP's own knowledge of the workplace; a consideration of the temporal relationship between the occurrence of problems and the stated pressures, events or changes at work; and ensuring that the patient's description of the injury and workplace environment corroborates with actual events (ie, plausibility).

### How can the condition be managed effectively to improve personal recovery or return to work?

For most patients with a work‐related MHC, their GP has a significant role in the recovery journey, including setting expectations for recovery, explaining and discussing potential treatment options, and identifying and collaborating with other key professionals with expertise in MHC who can provide the patient with the optimal care and management. Box 4 provides advice about key aspects of care that can enhance personal recovery at work or return to good and safe work in patients with a work‐related MHC.

Box 4 **Recommendations in response to the question “How can the condition be managed effectively to improve personal recovery or return to work?”**

**Table:** 

	Evidence level
Adopt a patient‐centred approach. Refer to existing high quality guidelines for the management of mental health conditions, while considering work‐related factors	Consensus‐based recommendation
In recognition of the health benefits of safe work and in regards to personal recovery, consideration should be given, when appropriate, to whether a patient can remain at or return to work (this may include transition back to work or work modification)	Consensus‐based recommendation
In patients with a secondary work‐related mental health condition, when the primary condition was a musculoskeletal injury, a general practitioner may consider work‐directed cognitive behavioural therapy	GRADE: Weak; Evidence: Moderate

GRADE = Grading of Recommendations Assessment, Development and Evaluation.

A patient‐centred approach involves addressing the clinical aspects of the illness, the patient's perceptions, beliefs and attitudes, and environmental factors that can promote or hinder recovery. It is important that patients provide consent to contact other health professionals, workplace representatives or other individuals, such as cultural consultants or family members, who can advocate for the patient's needs and concerns.

### Can the patient work in some capacity?

Engaging in good, safe and meaningful work has many benefits on health.^26^, ^27^ Benefits to a person's mental health alone include a greater sense of autonomy, improved wellbeing, improved recovery from MHCs, increased access to resources to cope with demands, enhanced social status and access to opportunities that stimulate personal development.^27^ As such, it is important that engagement in work is considered early and as part of any treatment and recovery plan. When a patient's workplace is likely to be safe and supportive, the strategy may involve continuing to work at the workplace, with possible adjustments made to duties, timings or other aspects of work. When a person is on sick leave, a transition to safe work may facilitate recovery. Box 5 outlines the range of patient and workplace factors that can assist the GP to determine whether a patient has the capacity to work.

Box 5 **Recommendations in response to the question “Can the patient work in some capacity?”**

**Table:** 

	Evidence level
A general practitioner should consider the following patient‐ and work‐related factors when determining whether a patient has the capacity to work:	
Patient‐related factors: ▶ severity of the mental health condition; ▶ presence of comorbidities; ▶ presence of sleep disturbance; ▶ higher conscientiousness pre‐injury; ▶ attitude towards work; ▶ patient motivation to work; ▶ work ability; ▶ personal circumstances (personal relationships, finances, housing arrangements, level of physical activity); and ▶ social deprivation (social or cultural disadvantage) Work‐related factors: ▶ work environment; ▶ GP's knowledge about the patient's workplace and its limitations; ▶ suitability of work; ▶ size of the workplace; ▶ conflicts with the patient's supervisor; ▶ ongoing work‐related stressors (eg, conflict with colleagues in the workplace); and ▶ availability of duties that are non‐stigmatising and, where possible, commensurate with the worker's level of experience and seniority	Consensus‐based recommendation
A GP should consider consulting with a workplace rehabilitation provider in order to make an assessment of the workplace environment	Practice point

The decision to recommend staying at work or returning to work is a balance of symptom management, consideration of the patient's beliefs and attitudes, and appropriateness of the workplace and work duties. When the GP identifies factors that are inhibiting the patient's return to work, the GP should aim to address these. For instance, if returning to work is likely to benefit the patient, but the patient is fearful of re‐injury,^28^ the GP can work with the patient and the employer to alleviate these concerns. Conversely, if the patient has the capacity to work but returning to work with the pre‐injury employer cannot be achieved, consider a work‐conditioning program with a similar organisation (if appropriate). When a patient has had a workers’ compensation claim accepted, the GP can request access to information or reports on the risks and possible return to work duties from the insurer or employer and use this information to determine if the patient can transition back to work.

The GP may wish to collaborate with other health professionals, such as an occupational physician or a workplace rehabilitation provider who may be a qualified rehabilitation counsellor, to assist in making an educated assessment of the workplace environment and the appropriateness of duties for the patient to ensure safety and continued recovery. Care should always involve the patient and, when appropriate, their advocates such as cultural consultants, community members or family members,^4^ and should focus on the patient's needs.

### What is appropriate communication with the patient's workplace?

Constructive communication between the GP and the patient's workplace can enhance the patient's recovery by enabling the patient to stay at or return to safe and meaningful work, or by identifying work factors that may hinder the patient's recovery and using a collaborative approach with the workplace to address these. Box 6 provides advice on best practice methods of communication between the GP and a patient's workplace to foster a collaborative patient‐centred approach for managing a work‐related MHC.

Box 6 **Recommendations in response to the question “What is appropriate communication with the patient's workplace?”**

**Table:** 

	Evidence level
A general practitioner should use telephone and/or face‐to‐face methods to communicate between a worker, supervisor, health care providers, union representatives and other disability management stakeholders	GRADE: Strong; Evidence: Moderate
A GP should consider using a trained workplace rehabilitation provider, if available, to coordinate and negotiate return to work among stakeholders	GRADE: Strong; Evidence: High
When discussing the care of a patient who has a work‐related mental health condition with their workplace, ensure that communication* maintains a focus on the workplace and on the worker's needs and functional capacities	Consensus‐based recommendation

GRADE = Grading of Recommendations Assessment, Development and Evaluation. *Communication between a GP and the patient's workplace should only occur with the patient's consent.

The GP can ensure that communications are safe and productive for the patient by:

- discussing their communication content with the patient before engaging with the patient's employer, with a focus on the workplace and the patient's needs and functional capacities;
- deciding with the patient who should be involved in the communication, including patient advocates (such as cultural representatives or family members);^4^
- carefully recording all communications, including phone conversations with employers, and providing written recommendations, after communication, to the patient and others that outlines work adjustments or considerations that need to be made; and
- engaging with other health professionals such as workplace rehabilitation providers or occupational physicians to aid the conversation.

For those patients who have a workers’ compensation claim accepted, case conferencing arrangements are now available and funded in most jurisdictions across Australia. GPs may consider using these services to support a timely and coordinated approach to return to work for the patient.

### What can a general practitioner do for a patient whose mental health condition is not improving?

Due to their complex biopsychosocial nature, MHCs can take months or years to resolve and continuing or new stressors may impede recovery. Box 7 provides advice on strategies to improve personal recovery in patients with MHCs that are not improving as anticipated.

Box 7 **Recommendations in response to the question “What can a general practitioner do for a patient whose mental health condition is not improving?”**

**Table:** 

	Evidence level
On the available evidence, there is no clear support for an intervention in a general practice setting to improve personal recovery or return to work in patients with a work‐related mental health condition who are not improving; therefore, there is an urgent need to promote research in this area	Recommendation for future research
In patients with a persistent mental health condition that has arisen out of work, a GP should: investigate the existence of continuing work‐related and non‐work‐related stressors that may contribute to delayed patient recovery and assist to address them; review the diagnosis and treatment plan to ensure that the patient is receiving optimal treatment; and adopt a patient‐centred collaborative care approach with relevant health professionals	Consensus‐based recommendation
When no work‐related or non‐work‐related stressors can be identified, and when persistent depression is present, a GP may consider the following evidence‐based approaches to treat the persistent depression: collaborative care between relevant health professionals for patients with persistent depression; and cognitive behavioural therapy as an adjunct to pharmacotherapy for patients with treatment‐resistant depression	GRADE: Weak; Evidence: High

GRADE = Grading of Recommendations Assessment, Development and Evaluation.

When investigating the existence of continued workplace stressors and revising the treatment plan, the GP may engage with the patient's workplace to investigate the stressors and advocate to the workplace on behalf of the patient to help manage the stressor. Case conferences are a useful method for discussing and addressing work‐related stressors. If the GP is not in a position to manage a work‐related stressor (eg, ongoing bullying), or if the patient does not consent for the GP to communicate with the workplace, the GP can, with the patient's consent, seek independent remediation to negotiate the changes that need to be made to ensure a safe return to work.

## Future research

Although we endeavoured to provide evidence‐based advice to address all the clinical questions, for some questions no reliable evidence could be identified. The guideline development group therefore recommended that further research investigating work‐related MHCs be undertaken. These areas are described in Box 8.

Box 8 **Recommendations for future research**

**Table:** 

Future research investigating work‐related mental health conditions (MHCs) should be undertaken on the following areas: population groups — culturally and linguistically diverse people, young people, people living in rural and remote Australia, and Aboriginal and Torres Strait Islander populations; an instrument to indicate the probability that an MHC has arisen out of work; interventions in the general practice setting to improve personal recovery or return to work in patients with a work‐related MHC; interventions in the general practice setting to manage comorbid substance misuse or addictive disorders; and interventions in the general practice setting to improve personal recovery or return to work in patients with a work‐related MHC who are not improving

In addition to the recommendations for future research, the guideline development group noted gaps in the evidence on the following areas:

- management strategies for work‐related MHCs that are feasible and acceptable for GPs to utilise, including special considerations for GPs practising in rural and remote Australia;
- evidence to describe the value of work participation for people with a work‐related MHC; and
- feasible tools and strategies that are validated for use in the general practice setting to support the diagnosis and management of acute stress disorder and adjustment disorder.

## Competing interests

The online Supporting Information includes the declaration of competing interests.

## Provenance

Not commissioned; not externally peer reviewed.

## Supporting information

Competing interests declaration
