# Supplementary material for: Diagnosing and managing work‐related mental health conditions in general practice: new Australian clinical practice guidelines
Source: Med J Aust. 2019 Jun 24;211(2):76–81. doi: 10.5694/mja2.50240 (PMC6852433; doi:10.5694/mja2.50240)
Supplement: Supplementary file 1 — Competing interests declaration [file MJA2-211-76-s001.pdf]

The Medical Journal of Australia

Appendix

This appendix was part of the submitted manuscript and has been peer reviewed.
It is posted as supplied by the authors.

Appendix to: Mazza D, Chakraborty SP, Brijnath B, et al. Diagnosing and managing work-related mental health conditions in general practice: new Australian clinical practice guidelines. *Med J Aust* 2019; 211. doi: 10.5694/mja2.50240.

Appendix A. Guideline Development Group Conflicts of Interest

Member	Position	Affiliation	Conflict of Interest
Prof Danielle Mazza	Content expert (Chair)	Monash University, Department of General Practice / General Practitioner	Research support (monetary support) – Research grant awarded by Steering Group member organisation ISCRR. The project ceased in 2016.
Dr Bianca Brijnath	Content expert	Monash University / National Aging Research Institute	Research support (monetary and non-monetary support) – Research grant awarded by Steering Group member organisation ISCRR. The project ceased 2016.<br>Paid work with WorkCover WA. Work ceased in 2015.<br><br>Intellectual property – Published paper with Work Safe Victoria, TAC and ISCRR. A potential conflict of interest was declared at Guideline Development Group Meeting #3 where this paper was discussed. The COI was managed with Dr Brijnath stepping out of the meeting room for the duration of the discussion.
Dr Samantha Chakraborty	Methodologist/ Project Manager	Monash University, Department of General Practice	None declared.
Ms Heather Nowak	Consumer	Mental Health Australia	None declared.
Dr Cate Howell	General practitioner	Royal Australian College of General Practitioners	None declared.
Dr Trevor Brott	General practitioner, content expert	Practicing General Practitioner	None declared.
Dr David Gras	Occupational physician	Royal Australian College of Physicians (Australasian Faculty of Occupational and Environmental Medicine) / Occupational Physician	Employment and consulting – Referral of cases for independent specialist medical assessment with Work Life Well. This position receives minor financial income <$5,000.
Dr Michelle Atchison	Psychiatrist	Royal Australian and New Zealand College of Psychiatrists	Employment and consulting – Chair of the South Australian Ministerial Advisory Committee reviewing the function and implementation of the new Return to Work Act. This is a gazetted position with remuneration.

Member	Position	Affiliation	Conflict of Interest
Prof Justin Kenardy	Psychologist	Australian Psychological Society	Employment and Consulting – Consulted on the NHMRC PTSD Guidelines with Phoenix Australia.<br><br>Research support (monetary support) – Received Motor Accident Insurance Commission funding for the RECOVER project. The project continues to be undertaken by Prof Kenardy at the RECOVER Research Centre at University of Queensland.
Ms Fiona Emery (meetings 1 and 2)<br>Mr Richard Buchanan (meeting 3 onwards)	State-based policy maker	Office of Industrial Relations – Queensland Government	None declared.<br><br>None declared.
Mr Seyram Tawia	National workers compensation scheme representative	Comcare	None declared.
